# Hemoglobin Decrease with Iron Deficiency Induced by Daclatasvir plus Asunaprevir Combination Therapy for Chronic Hepatitis C Virus Genotype 1b

**DOI:** 10.1371/journal.pone.0151238

**Published:** 2016-03-18

**Authors:** Nobuyuki Matsumoto, Hiroki Ikeda, Ryuta Shigefuku, Nobuhiro Hattori, Tsunamasa Watanabe, Kotaro Matsunaga, Tetsuya Hiraishi, Tomohiro Tamura, Yohei Noguchi, Yasunobu Fukuda, Toshiya Ishii, Chiaki Okuse, Akira Sato, Michihiro Suzuki, Fumio Itoh

**Affiliations:** Division of Gastroenterology and Hepatology, Department of Internal Medicine, St. Marianna University School of Medicine, 2-16-1 Sugao, Miyamae-ku, Kawasaki City, Kanagawa, 216–8511, Japan; National Taiwan University Hospital, TAIWAN

## Abstract

**Background:**

Decreased hemoglobin (Hb) level has been supposed to be a relatively rare side effect of a combination therapy against hepatitis C virus that consists of the NS5A inhibitor daclatasvir (DCV) and the NS3/4A protease inhibitor asunaprevir (ASV).

**Methods:**

The study was conducted in 75 patients with genotype 1b chronic hepatitis C virus infection who had started combination therapy with DCV and ASV at St. Marianna University School of Medicine Hospital between September 2014 and December 2014.

**Results:**

Among the patients examined, decreased Hb level by ≥1.5 g/dL from the values at treatment initiation was observed in 11 individuals. This was accompanied by decreased mean corpuscular volume, and iron and ferritin levels.

**Conclusions:**

These findings suggest that the mechanism of the phenomenon is caused by iron deficiency. The underlying mechanism and clinical impacts will need to be further examined.

## Introduction

A combination therapy against hepatitis C virus (HCV) that is composed of the NS5A inhibitor daclatasvir (DCV) and the NS3/4A protease inhibitor asunaprevir (ASV) has been commercially available in Japan since September 2014 as the first interferon (IFN)-free treatment. Elevated aspartate aminotransferase and alanine aminotransferase levels have been found in approximately 10% of cases and have attracted attention as adverse events of the combination therapy with DCV and ASV (DCV/ASV therapy). However, the overall occurrence of adverse events has been considered as having minor importance in comparison with those associated with the conventional IFN-based treatment. Findings from phase 3 clinical trials conducted in Japan and other countries have shown that the incidence of anemia during DCV/ASV therapy ranged from 2% to 3% [1,2]. However, our experience with a number of cases has shown that the incidence of decreased hemoglobin (Hb) levels during the treatment course is higher. Therefore, we selected patients who developed a mild decrease in Hb level during DCV/ASV therapy and examined their characteristics of the cases.

## Materials and Methods

### Patients

The study was conducted in 79 patients with genotype 1b chronic hepatitis C virus infection who were ineligible or contraindicated for continuous IFN treatment, or who were unresponsive to IFN treatment, and who received combination therapy with DCV and ASV at St. Marianna University School of Medicine Hospital between September 2014 and December 2014. Written informed consent was obtained from all the enrolled patients, and the protocol was approved by the institutional review board of St. Marianna University Hospital (approval No. 2806). Four patients were excluded because of treatment discontinuation due to early side effects in the early stages of treatment. Among the 75 remaining patients, 11 whose Hb levels during the period of treatment decreased by ≥1.5 g/dL from the concentrations measured at the beginning of treatment were included in the study. For the 75 patients who received combination therapy with DCV and ASV, with the exception of those who discontinued treatment in its early phase, the median duration (range) of the observation period was 13 weeks (8–18 weeks). The median age (range) at treatment initiation was 73 years (40–87 years). Men accounted for 24 patients, and women accounted for 51 patients. Patients with a fibrosis progression (FIB-4) index of ≥3.25 accounted for 48 cases, and those with mild fibrosis with a FIB-4 index of <3.25 accounted for 27 cases [3].

### Treatment protocol

In the combination therapy with DCV and ASV, DCV (Daclinza, Bristol-Myers Squibb K.K., Tokyo, Japan) was administered orally at 60 mg per dose, at a rate of one dose per day. ASV (Sunvepra, Bristol-Myers Squibb K.K.) was administered orally at 100 mg per dose, at a rate of 2 doses per day. For both medications, the treatment duration was 24 weeks.

### Laboratory assessment

Clinical parameters were measured by using standard laboratory techniques at a commercial laboratory (LSI Medience Co., Tokyo, Japan). Blood tests and measurements were performed at treatment initiation, at 1 week after treatment initiation, and subsequently, once every 2 weeks. For 11 patients whose Hb levels during the treatment period decreased by ≥1.5 g/dL from the values measured at treatment initiation, Hb level and mean corpuscular volume (MCV) were measured. Then, the values at treatment initiation were compared with those at the time when the Hb levels had decreased by ≥1.5 g/dL. In addition, stored serum samples collected at the time of each blood test were used for the measurement and comparison of serum iron levels, total iron-binding capacity, and serum ferritin concentrations. Additionally, we also investigated the Hb levels of the 11 patients during and after the DCV/ASV therapy.

### Statistical Analysis

A comparison between the two groups was conducted by using a paired *t* test against a group with normal distribution. In addition, for groups with non-normal distribution, comparisons were performed by using the Wilcoxon signed-rank test. A p < 0.05 was considered statistically significant.

## Results

Among the 75 patients examined, decreased Hb level by ≥1.5 g/dL from the values found at treatment initiation was observed in 11 individuals. The demographic characteristics of the patients are shown in Table 1.

**Table 1:** Comparison of Hb levels and MCVs and levels of iron metabolism markers between the baseline and the time of reduction in Hb level.

Case	Age (yrs)	Sex	The duration up to Reduction in Hb (wk)	Baseline					At the time of decrease in Hb by 1.5g/dl or more				
				Hb (g/dL)	MCV (fl)	Fe (μg/dL)	TIBC (μg/dL)	Ferritin (ng/ml)	Hb (g/dL)	MCV (fl)	Fe (μg/dL)	TIBC (μg/dL)	Ferritin (ng/ml)
1	73	F	4	12.6	92.3	77.7	566	20.0	10.9	82.1	32.6	559	6.1
2	70	F	14	11.9	87.8	79.2	478	20.8	10.1	80.9	30.8	454	14.8
3	80	F	2	13.4	91.0	62.5	411	52.4	11.5	89.6	46.1	356	32.0
4	82	M	6	9.6	85.4	21.3	490	26.7	8.1	83.5	21.8	451	19.9
5	83	M	12	12.4	93.3	113.2	456	32.2	10.9	85.6	51.7	470	16.6
6	72	F	8	13.6	96.2	100.9	394	27.1	12.1	94.6	83.8	381	18.0
7	76	F	11	13.2	98.2	82.0	425	239.4	11.7	97.6	99.5	344	64.5
8	64	F	4	14.6	98.9	78.0	355	39.9	12.9	97.2	114.4	376	23.8
9	62	F	10	12.4	83.2	38.4	412	7.9	10.6	82.9	20.2	375	6.4
10	82	F	8	10.2	89.6	62.5	522	29.3	8.7	85.5	23.9	491	16.0
11	76	F	6	13.1	88.8	61.0	471	19.4	11.4	87.3	23.4	502	10.8

**Fig 1:**
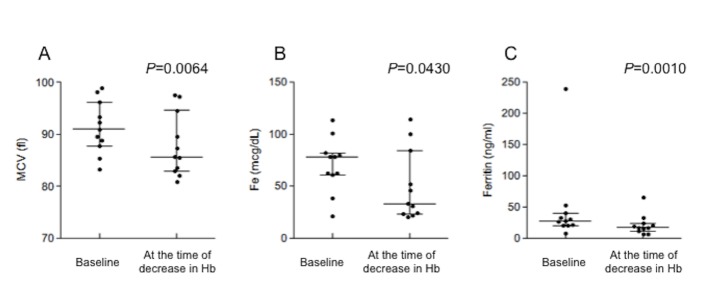
Change in (A) MCV, (B) serum iron level, and (C) ferritin level at the time of the decrease in Hb level Median values and interquartile ranges are indicated. A paired *t* test was performed for MCVs and serum iron levels, and the Wilcoxon signed-rank test was performed for the ferritin levels.

The median (range) of the period until the achievement of a decrease in Hb level of ≥1.5 g/dL was 8 weeks (2–14 weeks). The mean MCVs at baseline and at the time of the decrease in Hb level were 91.3 and 87.9 fl, respectively. These findings show a significant decrease in MCV at the time of the decrease in Hb level (Fig 1A). The mean serum iron levels at baseline and at the time of the decrease in Hb level were 70.6 and 49.8 μ/dL, respectively. A significant decrease in serum iron level was found at the time of the decrease in Hb level (Fig 1B). In addition, the median serum ferritin levels at baseline and at the time of the decrease in Hb were 27.1 and 16.6 ng/mL, respectively. A significant decrease in serum ferritin level was found at the time of the decrease in Hb level (Fig 1C).

**Fig 2:**
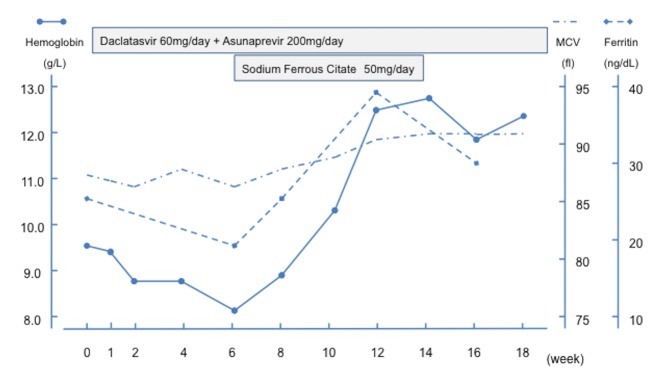
Clinical coarse of a representative case.

**Fig 3:**
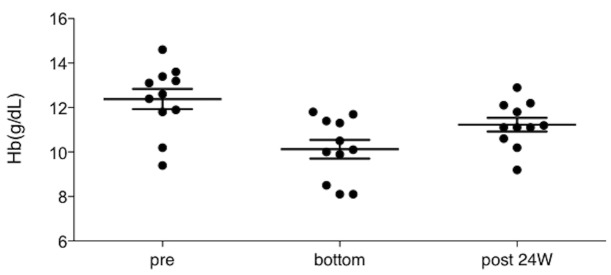
Recovery of decreased Hb level after DCV/ASV treatment Mean values and SDs of the Hb levels at each time point are indicated. pre: the baseline Hb level, bottom: the lowest Hb level during DCV/ASV treatment, post 24W: Hb levels at 24 weeks after the DCV/ASV treatment.

Fig 2. shows the changes in Hb level, MCV, and ferritin level during the treatment course, as found in the patient described as Case 4 in Table 1. Although the Hb level at treatment initiation was 9.6 g/dL, it had been consistent before the treatment. The Hb started to decrease in a stepwise manner after treatment initiation and reached as low as 8.1 g/dL at 6 weeks. Slightly reduced MCVs and decreased ferritin levels were also found. Examination for occult blood in stool showed a negative result. Based on the laboratory findings, iron deficiency was suspected, and small doses of an iron preparation were administered orally during continuation of DCV/ASV therapy. As a result, the Hb levels increased swiftly, as well as the MCVs and ferritin levels. Later, as the iron preparation was discontinued at 14 weeks, the ferritin levels decreased again. Reduced oral intake and other adverse events were absent during treatment; therefore, the same treatment has been maintained. Finally, we investigated the Hb levels of the 11 patients during and after the DCV/ASV therapy. All the patients had completed the treatment for hepatitis C virus infection. The changes in Hb level in the 11 patients during and after the treatment are shown in Fig 3. There is a tendency of recovery at 24 weeks after the treatment. This tendency supports the idea that the deceased Hb levels in the patients were related to the DCV/ASV treatment.

## Discussion

This study showed that in some patients, Hb level decreased during the course of DCV/ASV therapy, although this accounted for only a few cases. We examined Hb level, which decreased by ≥1.5 g/dL from the values at treatment initiation. In measurement conducted in the middle of ongoing treatment, 15% of the cases were consistent with the definition of decreased Hb level in this study. If the measurement had been performed throughout the entire treatment course, a larger number of patients might have shown a decrease in Hb level. The results of phase 3 clinical trials conducted in Japan and other countries of the world have shown that the incidence of anemia accounted for 2% to 3% of adverse events. This fact suggests that although DCV/ASV therapy causes only a mild decrease in Hb level, the phenomenon occurs at a relatively high frequency. Anemia has been reported to occur more frequently and more seriously in the treatment of hepatitis C virus infection with telaprevir and peg-IFN plus ribavirin than that with peg-IFN and ribavirin [4]. This result suggests that the direct action of anti-viral agents may induce a decrease in Hb levels. However, the contribution of telaprevir therapy in the treatment of anemia is unclear because of the influence of IFN and ribavirin, both of which could respectively induce anemia. This study showed that a decrease in Hb level can occur in DCV/ASV therapy, which does not contain IFN and ribavirin.

In addition, this study elucidates that the cases shared common features; that is, the fact that the phenomenon was accompanied by a decrease in MCV, and serum iron and serum ferritin levels. This shows that the decreased Hb level, which was caused by the DCV/ASV therapy, was the result of iron deficiency. In the case shown in Fig 2., ferritin and Hb levels increased rapidly as soon as the oral administration of an iron preparation was started. The ferritin level decreased again after the administration of the iron preparation was discontinued. This also supports the idea that the decreased Hb level is caused by iron deficiency [5]. During the course of the DCV/ASV therapy, 4 (cases 1, 4, 7, and 10) of the 11 cases were treated with oral iron supplementation. The results of their occult blood tests in stool were negative. None of them showed any symptoms related to decreased Hb level. All of these cases showed the similar clinical courses. Although the decreased Hb levels did not cause any symptoms in all the patients, 3 patients showed a decrease in Hb level of >3 g/dL from the baseline value. Thus, monitoring and relevant intervention may be necessary. In addition, we confirmed a tendency of the Hb levels to recover after DCV/ASV treatment completion, except for some protracted cases. Because the protracted cases were elderly patients, the protraction might be interpreted as an age-related phenomenon. None of the patients had any clinical symptoms and positive examination results for occult blood in stool. Thus, we believe that all of them will recover to their baseline status. The time of 24 weeks might be inadequate for old patients to recover. Further study is required concerning this point.

Serum ferritin levels indicate the amount of iron stored in the body [6]. Generally, patients with chronic liver disease often have higher serum ferritin levels [7]; however, for the majority of the patients in the group with decreased Hb level in this study, the ferritin levels at treatment initiation were within the normal range and were low. Iron overload has been indicated to aggravate chronic hepatitis infection [8], and a previous report showed the effectiveness of iron-restricted diet in such cases [9]. For this reason, an iron-restricted diet is generally recommended for patients with chronic hepatitis C virus infection in Japan. For people with poor iron storage, iron restriction is likely to cause iron deficiency. Therefore, when such treatment is introduced to patients with chronic hepatitis C virus infection, the iron restriction may need to be discontinued before initiating the treatment.

In the 11 cases that we experienced, the mechanism behind the iron deficiency remains unknown. In elderly subjects, iron deficiency is due to negative iron balance, and the underlying causes can be largely classified into 1) poor dietary intake, 2) loss from the digestive tract, and 3) malabsorption [10]. The 11 cases in this study showed no loss of appetite, suggesting that the iron intake did not decrease from the value before treatment initiation. In men or in postmenopausal women, acquired iron deficiencies, which develop within a relatively short period, are frequently due to an increased iron loss caused by blood loss resulting from gastrointestinal bleeding [11]. Neither apparent bleeding nor occult blood in stool was found in any of the 11 cases in this study, but detailed examination is necessary. In addition, DCV or ASV may also inhibit iron absorption. Other examples of drugs that inhibit iron absorption include proton pump inhibitors (PPI) [8] and tetracycline antibiotics [12]. Gastroesophageal reflux disease (5/11 cases) and osteoporosis (5/11 cases) were the most common morbidities in the 11 cases. PPI was the most frequently administered concomitant drug (5/11 cases). On the other hand, 6 cases showed decreased Hb level without PPI therapy, and 10 cases did not show decreased Hb level with PPI therapy. Taken together, in the present study, we could not identify any specific comorbidities and concomitant drugs that were related to the decreased Hb level during the DCV/ASV therapy.

## Conclusions

This study revealed that DCV/ASV therapy caused decreased Hb levels due to iron deficiency. However, the study was conducted in a small scale, and the measurements were conducted in the middle of an ongoing treatment. Hence, the underlying mechanism and the clinical impacts will need to be further examined.

## Abbreviations

HCV: Hepatitis C virus
DCV: Daclatasvir
ASV: Asunaprevir
Hb: Hemoglobin
MCV: Mean corpuscular volume
